# Clinical analysis on 430 cases of infantile purulent meningitis

**DOI:** 10.1186/s40064-016-3673-4

**Published:** 2016-11-21

**Authors:** Zhihui He, Xiujuan Li, Li Jiang

**Affiliations:** Department of Neurology, Children’s Hospital, Chongqing Medical University, 136 Second Zhongshan Road, Chonqqing, 400014 People’s Republic of China

**Keywords:** Infantile purulent meningitis, Clinical manifestation, Diagnosis

## Abstract

**Background:**

Purulent meningitis (PM) usually caused by a variety of pyogenic infection, is a kind of central nervous system infectious disease mostly common in children. It is easily misdiagnosed and its symptoms are varied. Excessive application of broad-spectrum antibiotics results in increased sickness and death of infants and young children. In this study, clinical data of 430 cases of PM in infants were analyzed to summarize the clinical experiences so as to achieve early diagnosis and early treatment of PM.

**Results:**

Male-to-female ratio was 1.61:1, and the median age of incidence was 0.42 years. May was the modal month of onset (11.7%). Main clinical manifestations were fever (89.3%), vomiting (67.2%), mental fatigue (62.1%), anterior fontanelle full/bulging/high tension (54.2%), convulsion (52.6%), and meningeal irritation sign (24.7%). Cerebrospinal fluid (CSF) bacterial culture was done for 420 cases, of which 1.2% cases were positive. Blood culture was done for 146 cases of which 15.1% were positive. 175 (40.7%) cases had complications, among which 133 cases (76.0%) were subdural effusion, 21 cases (12.0%) were epilepsy.

**Conclusion:**

Infantile PM is common in Spring, and May is the modal month of onset. The CSF/blood pathogen detection rate is very low and it is difficult to find evidence of cause. Fever, vomiting, mental fatigue and anterior fontanelle full/bulging/high tension, convulsion are the main clinical manifestations on which diagnosis depends. For those children diagnosed as PM and still having recurrent fever and prominent anterior fontanelle after treatment, clinicians should consider the probability of subdural effusion and treat them with brain imaging test on time.

## Background

Purulent meningitis (PM) usually caused by a variety of pyogenic infection, is a kind of CNS infectious disease mostly common in children, especially in infants and young children. It may leave sequelae and even lead to death (Bryan et al. 1990; Carroll and Carroll 1994; Feigin and Dodge 1976; Grimwood 2001; Nottidge 1985; Olanrewaju et al. 1991; Saez-Llorens and Mccracken 2003; Wang et al. 2015). PM is easily misdiagnosed because infants and young children lack the ability to describe their conditions, and its symptoms are varied. Excessive application of broad-spectrum antibiotics results in increased sickness and death of infants and young children (Carroll and Carroll 1994; Olanrewaju et al. 1991). At present in developed countries, the mortality rate is 5%, and 15% of patients have sequelae (De Jonge et al. 2010). To summarize the clinical experiences, so as to achieve early diagnosis and early treatment, clinical data of 430 cases of PM in infants admitted in our hospital between January 2004 and December 2013 are reported and analyzed below.

## Methods

### Case inclusion criteria

The golden diagnosis criteria of PM is to find the evidence for the existence of bacteria in CSF. However, this becomes difficult since excessive application of broad-spectrum antibiotics or limitations of bacteria detection technology, etc. Thus comprehensive analysis of the clinical data and experience on the clinical symptoms and signs are quite important to the diagnosis and treatment of PM. We collected and retrospectively analyzed the clinical data of PM in infants to achieve early diagnosis and early treatment. The clinical data of 430 cases of infantile PM admitted in Children’s Hospital of Chongqing Medical University were collected and all the selected cases were consistent with the inclusion criteria of PM (Hu and Jiang 2008): (1) age: 29 days after birth (We set this lower limit of age for excluding PM of newborns) to 3 years old; (2) clinical symptoms and signs of PM such as fever, vomiting, mental fatigue, anterior fontanelle full/bulging/high tension, convulsions, sleepiness, restlessness, irritability, meningeal irritation, coma, paralysis of limbs, headaches; (3) CSF routine and biochemical examinations show inflammatory changes: at least 3 features of the following: increase of WBCs (>500 × 10^6^/L), level of polymorphonuclear cells >50%, significant increase of protein content (>0.45 g/L), significant decrease of sugar (<2.4 mmol/L) or chloride content (<120 mmol/L); (4) CSF bacterial culture is positive or bacteria are found by Gram staining; (5) other intracranial infections can be ultimately excluded after hospital examinations and treatments. The patients of this group are diagnosed mainly based on items (1), (2), (3), (5), a few based on items (1), (2), (3), (4).

### Summary and analysis of clinical data

Detailed clinical data were collected and CSF/blood specimen samples of all patients were collected for inspection by conventional procedures within 24–48 h after hospitalization. The onset time, age, gender, main clinical manifestation, first CSF results, blood routine, blood electrolyte, blood culture, CSF culture, brain computed tomography (CT), magnetic resonance imaging (MRI), electroencephalograms (EEG), application of antibiotics and complications were recorded and analyzed.

### Statistical analysis

This study is largely descriptive. Data are presented as counts or percentages. Only descriptive statistics are presented.

## Results

### General information

In the 430 cases of PM, there were 265 males and 165 females; the male to female ratio was 1.61:1. The age of onset ranged from 29 days to 3 years, in which 29 days to 1 year, 1–2 years and 2–3 years accounted for 81.6% (351 cases), 15.4% (66 cases) and 3.0% (13 cases). The age of incidence peak was less than 1 year old, which accounted for 81.6%. And the median age of incidence was 0.42 years.

**Fig. 1:**
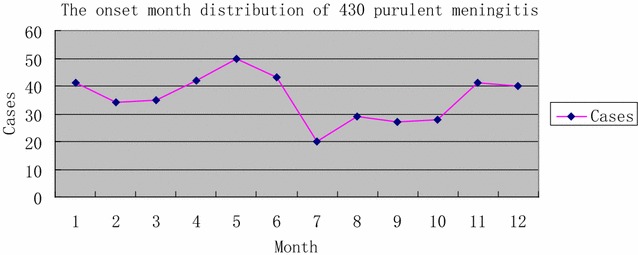
The onset month distribution of 430 purulent meningitis. Spring is the season of the highest incidence and winter is the second highest. Cases in May were the most frequent (11.7%)

During the past 10 years, cases in May were the most frequent (11.7%). In general, Spring is the season of the highest incidence and winter is the second highest (Table 1; Fig. 1).

**Table 1:** Distribution of 430 cases of purulent meningitis by month of onset

Month	1	2	3	4	5	6	7	8	9	10	11	12
Cases	41	34	35	42	50	43	20	29	27	28	41	40
Percentage (%)	9.5	7.9	8.1	9.8	11.7	10.0	4.7	6.7	6.3	6.5	9.5	9.3

Of these 430 cases, 229 (53.3%) came from rural areas and 201 (46.7%) from towns. There were 186 (43.2%) cases with less than 3 days’ time from onset to admission, 189 (44%) with time 3–10 days and 55 (12.8%) with time more than 10 days. Of the latter 55 cases, 48 (87.3%) were 29 days to 1 year of age. The length of hospital stay was from 3.5 h to 81 days, with a mean of 15.3 days.

In 225 (52.3%) cases, antibiotics had been used intravenously before admission; these included penicillin, ceftriaxone sodium, and cefotaxime sodium. Those without antibiotic treatment before admission accounted for 117 (27.2%) cases, and in 88 (20.5%) cases it was unknown whether antibiotics had been used or not.

### Clinical symptoms and signs

The main clinical symptoms of these 430 PM cases were in Table 2, where the most common clinical features were fever (384 cases, 89.3%), vomiting (289 cases, 67.2%), mental fatigue (267 cases, 62.1%), anterior fontanelle full/bulging/high tension (233 cases, 54.2%), convulsions (226 cases, 52.6%), sleepiness (162 cases, 37.7%), restlessness, irritability, (122 cases, 28.4%) and meningeal irritation (108 cases, 24.7%).

**Table 2:** Clinical manifestations of 430 cases of purulent meningitis

Clinical manifestations	Cases	Percentage (%)
Fever	384	89.3
Vomiting	289	67.2
Mental fatigue	267	62.1
Anterior fontanelle full/bulging/high tension	233	54.2
Convulsions	226	52.6
Somnolence	162	37.7
Restlessness/irritability	122	28.4
Meningeal irritation	108	24.7
Coma	62	14.4
Paralysis of limbs	28	6.5
Headaches	6	1.4

Not listed in Table 2 were five cases of jaundice, one case of ataxia. There were 45 cases (10.5%) of PM that were misdiagnosed in the early disease stages. All of the patients were between 29 days and 1 year. Of these, 30 cases were misdiagnosed as respiratory infections and gastroesophageal reflux, 12 cases misdiagnosed as febrile seizures and 3 cases misdiagnosed as sepsis.

### Laboratory tests

In the routine blood test for these 430 PM cases, there were 9 cases (2.1%) with WBCs < 4 × 10^9^/L, 118 cases (27.4%) between 4 × 10^9^ and 10 × 10^9^/L, 212 cases (49.3%) between 10 × 10^9^ and 20 × 10^9^/L, and 91 cases (21.1%) with WBCs > 20 × 10^9^/L. There were 280 cases (65.1%) in which the neutrophil proportion was greater than 50%.

There were 98 cases (22.8%) with blood sodium <135 mmol/L. The lowest level was 103.8 mmol/L. No hypernatremia was found.

The levels of WBCs, polymorphonuclear cells, glucose and proteins in CSF routine biochemical test for the 430 children with purulent meningitis are shown in Table 3. And the level of chlorides were 88.6–146.9 mmol/L, with the average 118.6 mmol/L.

**Table 3:** The CSF routine biochemical test results in 430 children with purulent meningitis

CSF routine biochemical test results		Cases	Percentage (%)
Level of WBCs	0–15 × 10^6^/L	49	11.4
	15 × 10^6^–100 × 10^6^/L	156	36.3
	100 × 10^6^–500 × 10^6^/L	141	32.8
	500 × 10^6^–1000 × 10^6^/L	54	12.5
	>1000 × 10^6^/L	30	7.0
Level of polymorphonuclear cells	>50%	187	43.5
	<50%	243	56.5
Level of glucose	<2.0 mmol/L	91	21.2
Level of proteins	<0.45 g/L	97	22.6
	0.45–1.0 g/L	166	38.6
	>1 g/L	167	38.8

Among the 430 cases of PM, 420 cases were tested for CSF smear (including bacteria, Cryptococcus and Mycobacterium tuberculosis). There were 3 positive cases (0.7%), where 2 cases were Gram-positive cocci and one case was Gram-negative bacilli. 420 cases were also tested for CSF bacterial culture. There were 5 positive cases (1.2%).

146 cases were tested for blood culture where 22 cases (15.1%) were positive. The results are shown in Table 4. The main component of the positive bacteria in the CSF and blood culture was Gram positive cocci, which had resistance rates of 85% to penicillin and 100% sensitivity to vancomycin. Gram negative cocci were generally sensitive to meropenem.

**Table 4:** The microorganisms from the positive cases of CSF and blood culture

Microorganisms	Cases (CSF)	Cases (blood)
*Streptococcus pneumoniae*	2	5
*β*-*Hemolytic streptococcus*	1	
*Enterococcus faecium*	1	2
*Escherichia coli*	1	3
*Staphylococcus aureus*		2
*Staphylococcus haemolyticus*		2
*Staphylococcus epidermidis*		1
*Klebsiella pneumoniae*		3
*Enterococcus gallinarum*		1
*Listeria* sp.		2
*Micrococcus luteus*		1

### Brain imaging examination

CT was performed in 312 cases, of which 45.2% were abnormal (Table 5). Brain MRI was performed in 186 cases, of which 65.1% were abnormal (Table 6).

**Table 5:** The CT results in 312 children with purulent meningitis

Brain CT results	Cases	Percentage (%)
Normal	171	54.8
Extracerebral space widened, subdural effusion	98	31.4
Local or diffuse lower density lesion	31	10.0
Ventricle plumped, ventriculomegaly	6	1.9
Cerebral edema	3	1.0
Hydrocephalus	2	0.6
Cerebral atrophy	1	0.3
Total cases	312	100

**Table 6:** The MRI results in 186 children with purulent meningitis

Brain MRI results	Cases	Percentage (%)
Normal	65	34.9
Extracerebral space widened, subdural effusion	57	30.6
Abnormal brain signals	33	17.7
Widened supratentorial ventricles	10	5.4
Delayed cerebral white matter myelinization	7	3.8
Abnormal development of pineal cyst, etc.	5	2.7
Hydrocephalus	5	2.7
Cerebral atrophy	2	1.1
Cerebral abscess	2	1.1
Total cases	186	100

### EEG

EEG were prepared for 302 cases, of which 49.3% were abnormal. In these abnormal cases, 108 cases (72.5%) had background slowing down and slow waves increasing, 33 cases (22.1%) had sharp wave/spike wave/sharp slow wave and 8 cases (5.4%) had low voltage performance.

### Treatment

Infants with PM were empirically treated with penicillin plus trisubstituted cephalosporin after admission. If it was ineffective, other antibiotics were used according to experience or pathogenic results. Ceftizoxime, ceftazidime and ceftriaxone were used the most in these cases with 249 cases (57.9%), 82 cases (19.1%), and 64 cases (14.9%), respectively. During the treatment, 84 cases (19.5%) were switched to trisubstituted cephalosporin plus vancomycin. Another 28 cases (6.5%) were treated with meropenem, which was commonly used in combination with vancomycin and trisubstituted cephalosporin. 12 cases were treated immunoglobulin 1–2 g/kg.

### Outcome

Of the 430 patients with PM, 298 (69.3%) were cured, 113 (26.3%) improved, 16 (3.7%) self-discharged from hospital and 3 (0.7%) died. On the other hand, 175 (40.7%) cases had complications, among which 133 cases (76.0%) were subdural effusion, 21 cases (12.0%) were epilepsy, 7 cases (4.0%) were hydrocephalus, 5 cases were injury of facial nerve, 3 cases were ependymitis, 3 cases were alimentary tract hemorrhage, 2 cases were encephalopyosis and 1 case was pleural effusion.

There was 1 relapsed case of PM, a boy of 2 years and 8 months old. The inducements of relapse were otitis media and mastoiditis and the legacy of severe deafness.

## Discussion

PM is one kind of common serious intracranial infectious disease of children that usually occurs in infancy because of their incomplete development and less resistance. In this group of 430 PM cases, male patients are more than female (1.61:1) where 229 cases (53.3%) are from rural areas and 201 cases (46.7%) from towns. On the whole of the 10 years, cases in May are the most common (11.7%) among each month. Spring is the season of the highest incidence and winter is the second. The onset severity is different between infants. These data showed that there were 48 cases (87.3%) aged 29 days to 1 year among the 55 cases who went to hospital more than 10 days after occurrence. Analysis was also related to the following reasons: the anterior fontanelle was not yet closed, there were symptoms of intracranial hypertension, signs of meningeal irritation appeared later and the patients’ occult condition was difficult to detect.

In China, more than two third of PM cases in children are caused by *Meningococcus*, *Streptococcus pneumoniae*, *Bacillus influenzae* (Hu and Jiang 2008). In recent years, due to the widely and timely application of antibiotics, some pyogenic bacterial infections have been controlled in time, which reduced the incidence rate of PM. However, more and more unreasonable and irregular applications of antibiotics have increased the atypical features of PM, which made it difficult to seek pathogenic evidence.

This study shows that 52.3% (225/430 cases) of diseased children used antibiotics before going into hospital, while 20.5% (88/430 cases) of which were unknown whether used or not. In 420 cases of this group, CSF smears (including bacteria, Cryptococcus sp. and tuberculosis bacilli smear) were done, and in 420 cases CSF cultures were done; the result of the latter was five positive cases (1.2%), which is much lower than in previous reports (Johnson et al. 2007; Mylonakis et al. 1998; Scheld et al. 1997; Tunkel 2001). Of the 146 cases where blood culture was done, only 22 cases (15.1%) showed positive. Almost all bacteria on clinical application of antibiotics can cause drug resistance, which becomes more and more serious (Deasy 2009; Mcdonald 2006). In recent years, it has been found that pneumococcal not only has a resistance rate of 45% against penicillin, but also has a resistance rate against third generation cephalosporins of about half (El Bashir et al. 2003). The positive bacteria in CSF and blood culture tests were mainly Gram positive coccus with drug resistance rate 85% to penicillin and sensitive rate 100% to vancomycin. Gram negative coccus is generally sensitive to meropenem.

In this group of 430 PM cases, the positive ratio in CSF examinations was high while the cases with the whole typical features of CSF were rare. In the CSF examinations, there were 54 cases (12.5%) with WBC level 500 × 10^6^ to 1000 × 10^6^/L, 30 cases (7.0%) with WBC level over 1000 × 10^6^/L, 187 cases (43.5%) with polykaryocyte over 50%, 91 cases (21.2%) with glucose under 2.0 mmol/L and 167 cases (38.8%) with protein more than 1.0 g/L. This analysis is also related to the case that most of the patients used antibiotics before going into hospital. Therefore, for the patients without typical clinical features of CSF, comprehensive analysis combining with fully clinical data should be done to avoid misdiagnosis and missed diagnosis.

The main clinical manifestations of these cases were fever (89.3%), vomiting (67.2%), mental fatigue (62.1%), meningeal irritation (24.7%), anterior fontanelle full/bulging/high tension (54.2%) and convulsions (52.6%). These clinical manifestations suggest that, besides knowing the medical history provided by the guardians of those infants and children who come to hospital because of fever, clinicians should strengthen the clinical observation and look for clues in the early stages of the disease among the symptoms of vomiting, mental fatigue, restlessness and increased sleep. This study showed that the incidence of convulsions was 52.6%, which was lower than what was reported in the previous reports (Mcdonald 1972).

This analysis was related to the overlooked slight convulsions of infants, which prompted the clinicians to check the body carefully. There were 45 cases (10.5%) misdiagnosed in the early disease stages. They were all 29 days to 1 year old, which suggested that full observation and analysis and timely examinations such as CSF test are more needed for the early diagnosis and timely treatment of infants (Hoque et al. 2006).

In the 430 cases, 175 cases (40.7%) had complications. Among these cases of complications, there were 133 cases (76.0%) of subdural effusion, 21 cases (12.0%) of seizures, 7 cases (4.0%) of hydrocephalus. All these cases with complications were found through brain imaging test (CT or MRI) to have widened brain extracellular space or subdural hydroma. Thus, for those children diagnosed as PM and still having recurrent fever and prominent anterior fontanelle after treatment, clinicians should consider the probability of subdural effusion and treat them with brain imaging test on time.

Besides penicillin, 249 cases (57.9%) were treated with ceftizoxime, 82 cases (19.1%) were treated with ceftazidime and 64 cases (14.9%) were treated with ceftriaxone. During the treatment, 84 cases (19.5%) were switched to use vancomycin plus the third generation cephalosporins, and 28 cases (6.5%) were treated with meropenem. The cure rate of the 430 cases was 69.3% and the improvement rate was 26.3%, suggesting that this group of patients were still sensitive to the third generation cephalosporins which could be used as the empirical therapy of PM.

## Conclusions

Infantile PM is common in Spring. And May is the modal month of onset. The CSF/blood pathogen detection rate is very low and it is difficult to find evidence of cause. This study revealed that Fever, vomiting, mental fatigue and anterior fontanelle full/bulging/high tension, convulsion are the main clinical manifestations on which diagnosis depends. For those children diagnosed as PM and still having recurrent fever and prominent anterior fontanelle after treatment, clinicians should consider the probability of subdural effusion and treat them with brain imaging test on time.

## Abbreviations

PM: purulent meningitis
CNS: central nervous system
CSF: cerebrospinal fluid
CT: computed tomography
MRI: magnetic resonance imaging
EEG: electroencephalograms
